# A Machine Learning Study on Internal Force Characteristics of the Anti-Slide Pile Based on the DOFS-BOTDA Monitoring Technology

**DOI:** 10.3390/s22062085

**Published:** 2022-03-08

**Authors:** Chaoqun Wei, Qinglu Deng, Yueming Yin, Mengyao Yan, Meng Lu, Kangqing Deng

**Affiliations:** Faculty of Engineering, China University of Geosciences, Wuhan 430074, China; weixiaosongchaoqun@cug.edu.cn (C.W.); gragrousmoon@gmail.com (Y.Y.); yanmengyao@cug.edu.cn (M.Y.); lumeng@cug.edu.cn (M.L.); dengkangqing7913@cug.edu.cn (K.D.)

**Keywords:** anti-slide pile, distributed optical fiber sensing technology (DOFS), BP neural network, calculation of bending moment

## Abstract

Long-term monitoring of constructed anti-slide piles can help in understanding the processes by which anti-slide piles are subjected to the thrust of landslides. This paper examined the landslide control project of Badong No. 3 High School. The internal force of an anti-slide pile subjected to long-term action of landslide thrust was studied by Distributed Optical Fiber Sensing (DOFS) technology. The BP neural network was used for model training on the monitored strain values and the calculated bending moment values. The results show the following: (1) The monitoring results of the sensor fibers reflect the actual situation more accurately than steel rebar meters do and can locate the position of the sliding zone more accurately. (2) The bending moments distributed along the anti-slide pile have staged characteristics under the long-term action of landslide thrust. Three stages can be summarized according to the development trend of the bending moment values. These three stages can be divided into two change periods of landslide thrust. (3) The model produced by the BP neural network training can predict the bending moment values. In this paper, the sensing fibers monitoring over a long time interval provides a basis for long-term performance analysis of anti-slide piles and stability evaluation of landslides. Using the BP neural network for training relevant data can provide directions for future engineering monitoring. More novel methods can be devised and utilized that will be both accurate and convenient.

## 1. Introduction

Landslides are a geological hazard that is also a worldwide issue. Each year the economic losses caused by landslides reach hundreds of billions of dollars [1]. It is thus essential that landslides be effectively monitored and controlled. Anti-slide piles are a key element in landslide control and monitoring, the subject of increasing numbers of instruments and theories [2,3,4]. Anti-slide piles can transfer the thrust and shear force behind them from the sliding body to a stable layer such as bedrock, stabilizing a landslide. Many researchers [5,6,7] have installed sensors on anti-slide piles to collect dynamic data and study the long-term performance of anti-slide piles under the action of landslide thrust. Some studies [8,9] have used deformation monitoring to show landslides’ long-term and seasonal impact. It has also been shown that anti-slide piles can weaken the role of the sliding body behind them. Other researchers [10,11,12,13] have described the processes involved in stress variations of anti-slide piles through physical model tests and have explained the evolutionary process of the displacement and deformation of landslides. When used as a primary treatment measure, anti-slide piles make costs easy to control, and their effectiveness in controlling landslides is widely recognized by many experts. Accordingly, anti-slide piles or schemes are often combined with other methods of landslide engineering [14,15,16,17].

Analyzing the mechanical properties of anti-slide piles through data monitoring can provide a basis for evaluating landslide stability. At present, the main methods used in landslide monitoring [18,19] are total station, inclinometer, steel rebar meter, and level instrument—all of which are easy to use and understand. However, all are also point-based monitoring methods, posing a certain risk of detection failure. For this reason, it is impossible to accurately monitor the data changes associated with the required parts. Most monitoring sensors must be buried in anti-slide piles or the surrounding soil. Concrete, groundwater, and soil can disturb or corrode sensors, degrading their long-term monitoring performance. Moreover, the data collected by most point monitoring sensors are incomplete in their depth or time span. As a result, certain difficulties arise in analyzing anti-slide piles’ internal force and bending performance. Some researchers [20,21,22,23] have deployed monitoring sensors on anti-slide piles, including point-type and quasi-distributed ones. Through data collection and analysis, they have discovered that quasi-distributed monitoring results are more reliable and of more practical significance, allowing monitoring of and warning regarding landslides. However, quasi-distributed and point-based monitoring methods provide incomplete data acquisition and cannot reflect the actual force of anti-slide piles. DOFS technology has become one of the most promising monitoring technologies owing to its fully distributed system, small size, corrosion resistance, and anti-electromagnetic interference [24,25,26,27]. It can collect information such as strain and temperature at all points along the optical fiber in a structure. The development and application of DOFS has produced many research findings globally, with its application range expanding to many fields [28,29,30]. The optical fiber is arranged in anti-slide piles so that the collected optical fiber data reflect the force of the anti-slide pile as much as possible. Internal force analysis of anti-slide piles using optical fiber monitoring data has verified the long-term effectiveness of anti-slide pile management and the superiority of DOFS.

In recent years, the study of machine learning has intensified. This artificial intelligence-based approach uses a series of data for self-learning and training, producing a model that describes the data series. It provides a training model for continuous learning [31,32]. In the field of machine learning, the artificial neural network is a statistical training model inspired by the biological neural network [33,34]. It is of practical significance to combine the machine learning method with monitoring and predicting landslide anti-slide piles. In this paper, the strain and temperature data collected by the distributed sensing fibers and the steel rebar meters in the anti-slide pile are analyzed. The bending moment values distributed along the depth of the anti-slide pile are calculated and analyzed. The strain change values of the distributed sensing fibers and the calculated bending moment values are then taken, respectively, as the sample’s input and output values for model training. The BP neural network model obtained through data training can predict internal force and shed light on the long-term performance and stability of the anti-slide pile.

## 2. Case Study

### 2.1. Engineering Situation

**Figure 1:**
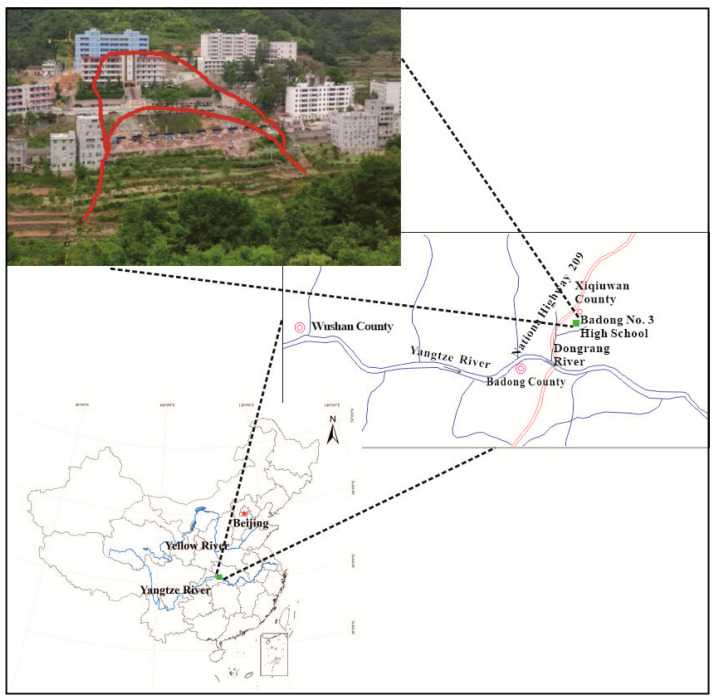
Geographical location of Badong No. 3 Senior High School.

**Figure 2:**
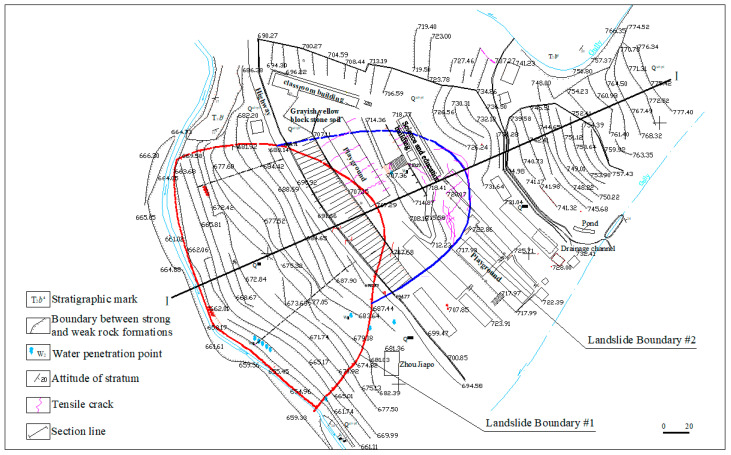
Plane diagram of landslides.

Badong County is located in southwest Hubei Province, China. Badong No. 3 Senior High School is located in Xiqiuwan Township, on the bank of the Xiaoxigou River, a tributary of Dongrang River, itself a first-level tributary of the north bank of the Yangtze River, as shown in Figure 1. The high school is situated on a landslide body (hereinafter referred to as the Sanzhong landslide). It developed on the early debris flow accumulation body and belongs to the Quaternary accumulation landslide. The landslide is intermittently active, with an occurrence in April 2002. The trailing edge of the sliding body is located in the playground at an elevation of 707 m. The front edge extends to the side of the Xiaoxigou River at an elevation of about 660 m. The main sliding direction is southwestward with 150 m long. The landslide resembles an inverted bowl, with an area of about 2.26 × 10^4^ m^2^ and a volume of about 30 × 10^4^ m^3^. The landslide area within this range is classified as landslide area #1. In July and August 2012, the sliding deformation area of the landslide increased, with more cracks appearing at the back edge of the original landslide, to an elevation of 730 m, due to rainfall and the traction of the landslide. According to the survey results, the landslide deformation was still developing, which seriously threatened the school’s main buildings. Since this activity, the area of the landslide is about 3.15 × 104 m^2^ and the volume about 60 × 10^4^ m^3^. The newly expanded landslide area is divided into landslide area #2. The plane morphologies of landslide areas #1 and #2 are shown in Figure 2.

**Figure 3:**
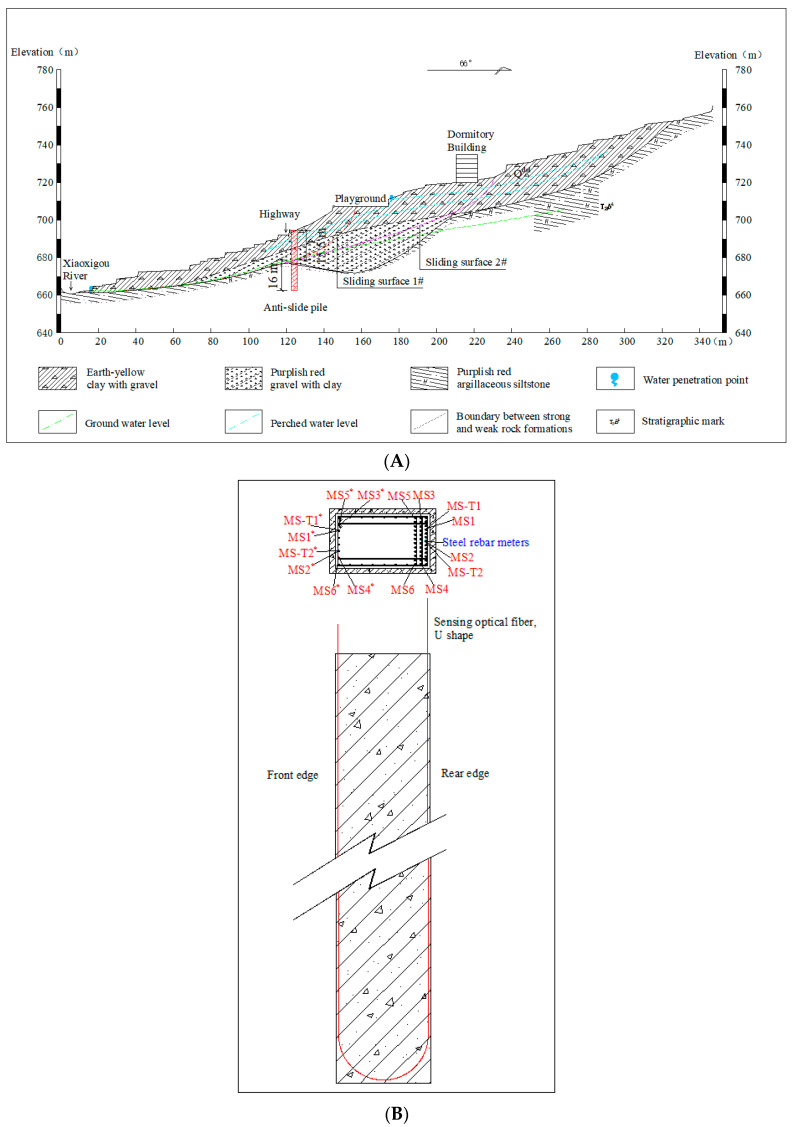
Vertical profile diagram (I-I) of landslide engineering and layout diagram of sensors and anti-slide pile. (**A**) Vertical profile diagram (I-I) of landslide engineering and anti-slide pile; (**B**) Layout diagram of optical fiber and steel rebar meters.

After investigation and study, an anti-slide pile protection measure was proposed to control the Sanzhong landslide. Before beginning the project, the researchers designed a monitoring scheme featuring distributed sensing fibers and steel rebar meters. Then the long-term anti-sliding performance of the anti-slide pile and the superiority of DOFS were evaluated using the collected data. According to the study, the sensing fibers were arranged in the anti-slide pile with a pile length of 31.5 m, as shown in Figure 3, with the loaded section 15.5 m and the embedded section 16 m. The plane dimension of the anti-slide pile was 2.0 × 3.5 m, and C30 concrete was used for on-site pouring. The anti-slide pile construction includes the following processes: construction preparation, pile hole excavation, groundwater treatment, wall protection, reinforcement cage production and installation, concrete perfusion, concrete maintenance, etc. The sensing fibers were bound along the tensioned side (rear edge) of the anti-slide pile’s main steel bars, passed through the bottom of the pile, and finally came out from the compressed side (front edge) of the anti-slide pile’s main steel bars in a U shape, as illustrated in Figure 3B. Considering that three rows of tensile steel bars with different lengths were arranged inside the anti-slide pile under differing stress conditions, a total of 8 sensing optical fiber loops were laid in the anti-slide pile schematically, as illustrated in Figure 3. The DOFS system featured 6 strain optical fiber loops (MS1, MS2, MS3, MS4, MS5, and MS6) and 2 temperature fiber loops (MS-T1 and MS-T2). It is worth noting that MS1, MS4, and MS-T2 were damaged in the concrete pouring process, but this does not affect the calculation and analysis of optical fiber data in the follow-up work and will not substantially affect the research results.

**Figure 4:**
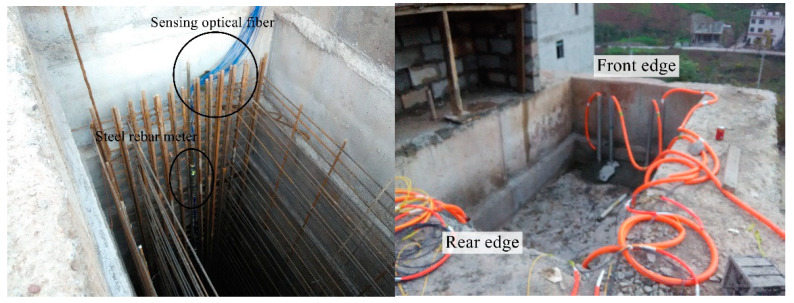
Site construction picture of the anti-slide pile.

The steel rebar meters were arranged on the tension side of the anti-slide pile and tied to the same reinforcement bar with MS2 and MS-T2, as shown in Figure 3. The steel rebar meters were welded to the reinforcement bar. A total of 16 steel rebar meters were laid with 2 m vertical spacing. The steel rebar meters were numbered from the pile bottom to the pile top. Steel rebar meter #1 was 0.5 m from the pile bottom, and steel rebar meter #16 was 1 m from the pile top. The field layout of the optical fiber and point sensor is shown in Figure 4.

The anti-slide pile was completed on 16 November 2015, with initial data gathering of the sensor fibers and steel rebar meters following on 21 November 2015. Data were collected 11 times for sensor optical fibers and steel rebar meters between November 2015 and March 2016. In September, October, and November of 2019, it was found that the steel rebar meters could no longer capture data, although the sensing fibers could still be used to collect data. Accordingly, the data of MS2, MS3, MS5, MS6, and MS-T1 are mainly analyzed, with the data from steel rebar meters compared with the corresponding fiber data.

### 2.2. Brillouin Fiber Technology and BP Neural Network

#### 2.2.1. Principles of BOTDA Monitoring

This paper used Pulse-PrePump Brillouin Optical Time Domain Analysis (PP-BOTDA) to collect and analyze data from sensing fibers. The spatial resolution of PPP-BOTDA can reach 5 cm, with a strain accuracy and range of 7.5 *με* and −30,000 *με* to +40,000 *με*, respectively. The data collected by this technology are the strain and temperature information reflected by the sensing fiber under the influence of external factors. According to the basic principle of stimulated Brillouin [35], the fiber’s frequency has a good linear relationship with the strain and temperature information in the region,

(1) $$\nu_{B}\left(\varepsilon ,T\right)=\frac{2n}{\lambda }\sqrt{\frac{\left(1-\mu \right)E}{\left(1+\mu \right)\left(1-2\mu \right)\rho }}$$

where $\nu_{B}\left(\varepsilon ,T\right)$ is the central frequency of the fiber affected by strain and temperature; n the refractive index of the fiber; λ the wavelength of the incident light; E Young’s modulus; μ Poisson’s ratio; and ρ fiber density, with n, E, μ, and ρ changing with changes in ε and T.

Equation (1) is expanded according to Taylor’s formula when ε and T are 0, respectively, in which high-order terms with extremely small magnitudes are ignored so that

(2) $$\nu_{B}\left(\varepsilon ,T\right)=\nu_{B}\left(0,T\right)\left(1+\left(\frac{\partial n}{\partial \varepsilon }n^{-1}+\frac{1}{2}\frac{\partial E}{\partial \varepsilon }E^{-1}-\frac{1}{2}\frac{\partial \mu }{\partial \varepsilon }\mu \left(\mu -2\right){\left(1-\mu^{2}\right)}^{-1}{\left(1-2\mu \right)}^{-1}-\frac{1}{2}\frac{\partial \rho }{\partial \varepsilon }\rho^{-1}\right)\Delta \varepsilon \right)$$

(3) $$\nu_{B}\left(\varepsilon ,T\right)=\nu_{B}\left(\varepsilon ,0\right)\left(1+\left(\frac{\partial n}{\partial T}n^{-1}+\frac{1}{2}\frac{\partial E}{\partial T}E^{-1}-\frac{1}{2}\frac{\partial \mu }{\partial T}\mu \left(\mu -2\right){\left(1-\mu^{2}\right)}^{-1}{\left(1-2\mu \right)}^{-1}-\frac{1}{2}\frac{\partial \rho }{\partial T}\rho^{-1}\right)\Delta T\right)$$

where $\frac{\partial n}{\partial \varepsilon }n^{-1}+\frac{1}{2}\frac{\partial E}{\partial \varepsilon }E^{-1}-\frac{1}{2}\frac{\partial \mu }{\partial \varepsilon }\mu \left(\mu -2\right){\left(1-\mu^{2}\right)}^{-1}{\left(1-2\mu \right)}^{-1}-\frac{1}{2}\frac{\partial \rho }{\partial \varepsilon }\rho^{-1}$ and $\frac{\partial n}{\partial T}n^{-1}+\frac{1}{2}\frac{\partial E}{\partial T}E^{-1}-\frac{1}{2}\frac{\partial \mu }{\partial T}\mu \left(\mu -2\right){\left(1-\mu^{2}\right)}^{-1}{\left(1-2\mu \right)}^{-1}-\frac{1}{2}\frac{\partial \rho }{\partial T}\rho^{-1}$ are constant terms about ε and T, respectively, when ε and T represent zero strain and zero temperature. They are abbreviated as $\frac{\partial \nu \left(\varepsilon ,T\right)}{\partial \varepsilon }$ and $\frac{\partial \nu \left(\varepsilon ,T\right)}{\partial T}$. Combining them with Equations (2) and (3) produces

(4) $$\nu_{B}\left(\varepsilon ,T\right)=\nu_{B}\left(\varepsilon_{0},T_{0}\right)+\frac{\partial \nu \left(\varepsilon ,T\right)}{\partial \varepsilon }\Delta \varepsilon +\frac{\partial \nu \left(\varepsilon ,T\right)}{\partial T}\Delta T$$

The Brillouin frequency changes when the fiber is subjected to force and temperature, which all cause strain variation at the corresponding position. These processes are all reflected by Equation (4). Before data acquisition, the appropriate parameters are set, and the corresponding microstrain and temperature values can be obtained through signal transmission and calculation of the instrument.

#### 2.2.2. BP Neural Network Algorithm

The BP neural network is a branch of machine learning that uses activation functions, weight values, network hierarchy structures, and many nodes to simulate the neurons and synapses of the biological brain [36]. It builds a corresponding model after training on sample data sets to predict output values. The BP neural network framework can be described as

(5) $${\hat{y}}_{j}=f\left(XW\right)$$

(6) $$X=\left[x_{1},x_{2},x_{3},\ldots \ldots ,x_{n}\right]$$

(7) $$W={\left[w_{i1},w_{i2},w_{i3},\ldots \ldots ,w_{in}\right]}^{T}$$

where ${\hat{y}}_{j}$ represents the output value; f the activation function; X the row vector of input values containing $x_{1},x_{2},x_{3},\ldots \ldots ,x_{n}$; and W the column vector of weight values containing $w_{i1},w_{i2},w_{i3},\ldots \ldots ,w_{in}$.

The output value is compared with the target value when an output value is obtained. If the error was greater than the set value, the algorithm would use error backpropagation to update the weight value [37]. The updated weight value will then be used to update the output value through the activation function again, obtaining the new output value,

(8) $$\delta_{j}=\left|{\hat{y}}_{j}-y_{j}\right|$$

(9) $$W_{x}{}^{updated}={\left[w_{\left(x1\right)i}+\eta \delta_{1}x_{i}\frac{df_{i}}{dy_{j}},w_{\left(x2\right)i}+\eta \delta_{2}x_{i}\frac{df_{i}}{dy_{j}},w_{\left(x3\right)i}+\eta \delta_{3}x_{i}\frac{df_{i}}{dy_{j}},\ldots \ldots ,w_{\left(xn\right)i}+\eta \delta_{j}x_{i}\frac{df_{i}}{dy_{j}}\right]}^{T}$$

(10) $$W_{y}{}^{updated}={\left[w_{i1}+\eta \delta_{1}y_{j}\frac{df_{i}}{dy_{j}},w_{i2}+\eta \delta_{2}y_{j}\frac{df_{i}}{dy_{j}},w_{i3}+\eta \delta_{3}y_{j}\frac{df_{i}}{dy_{j}},\ldots \ldots ,w_{in}+\eta \delta_{j}y_{j}\frac{df_{i}}{dy_{j}}\right]}^{T}$$

(11) $${\hat{y}}_{j}^{updated}=f\left(net_{j}\right)=f\left(XW^{updated}\right)$$

where $\delta_{j}$ represents the absolute error between the output value and the target value; $y_{j}$ the target value; $W_{x}{}^{updated}$ the weight accompanying the update of the input layer; $W_{y}{}^{updated}$ the weight accompanying the update of the hidden layer; η the learning rate; $\frac{df_{i}}{dy_{j}}$ the descending gradient; ${\hat{y}}_{j}^{updated}$ the updated output value; and $net_{j}$ the neural network framework.

The BP neural network algorithm continuously updates the weight value, with new output values obtained as the weight values are updated. Finally, the BP neural network algorithm model can be output when the output mean square error and performance are at their minimum, and the required accuracy is achieved.

### 2.3. Analysis of Monitoring Data

#### 2.3.1. Analysis of Strain and Temperature Data for BOTDA Technology

The strain and temperature of the anti-slide pile were monitored using DOFS-BOTDA technology. The sensing fibers used to collect strain and temperature comprise steel strands and sheaths of different strengths. Thus, the so-called strain data in this paper are simultaneously affected by deformation and temperature changes of the anti-slide pile, whereas the temperature data are affected only by temperature changes.

**Figure 5:**
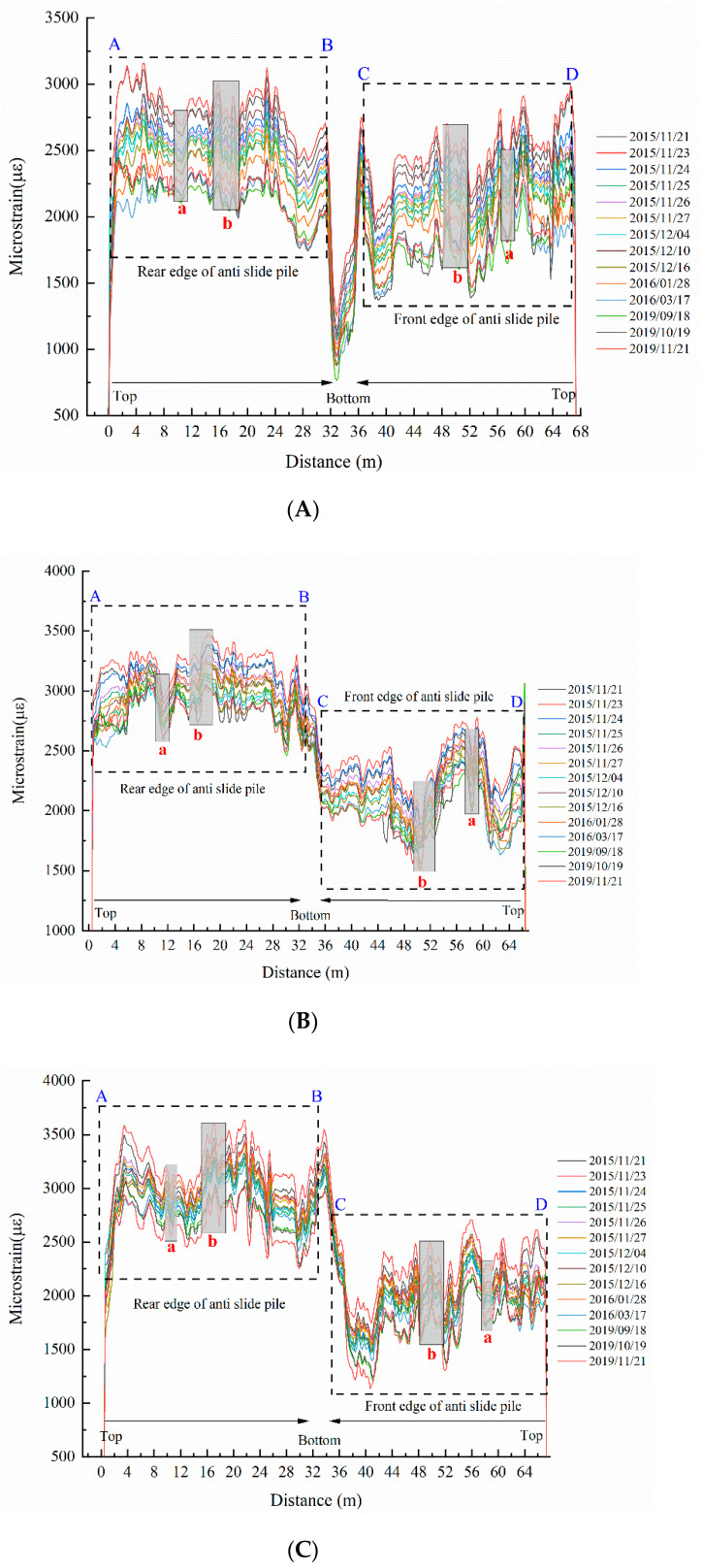

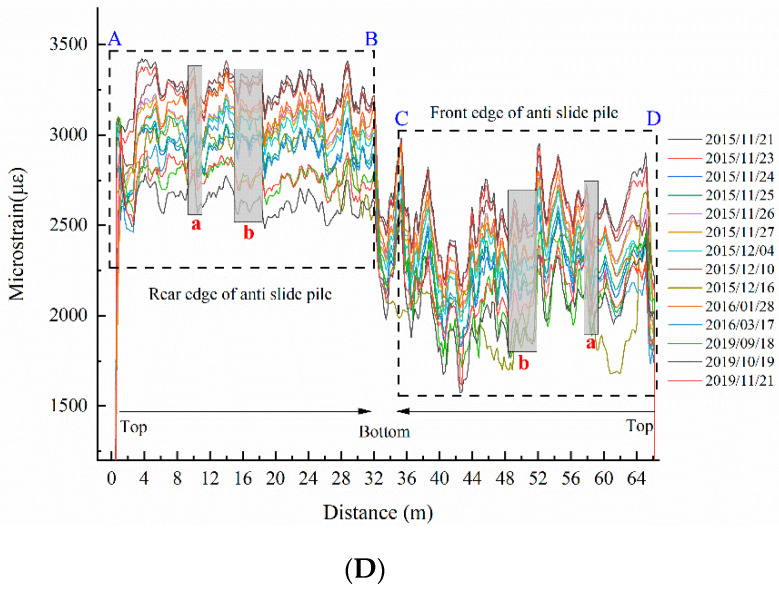
Strain curves diagrams of MS2, MS3, MS5, and MS6 for different periods. (**A**) Strain curves diagram of MS2; (**B**) Strain curves diagram of MS3; (**C**) Strain curves diagram of MS5; (**D**) Strain curves diagram of MS6. (In this figure, a represents optical data of the boundary between earth-yellow clay with gravel and purplish-red gravel with clay. Because the boundary between the loaded section and embedded section of the anti-slide pile is close to the boundary between strong and weak rock formations, b represents these two boundaries optical data and their adjacent optical data. The blue A and B represent the top and bottom of the rear edge of anti-slide pile, respectively; the blue C and D represent the bottom and top of the front edge of anti-slide pile, respectively).

The forces’ forms, magnitudes, and acting processes on the tensile side and the compressed side differ when anti-slide piles play their roles. Affected by these, two different regions can be clearly observed in the strain curves, as shown in Figure 5. It can be clearly distinguished that part A~B is the tension side (rear edge) of the anti-slide pile, and part C~D is the compression side (front edge) of the anti-slide pile.

According to Figure 3, different rock formations are boundaries in the area where the anti-slide pile sits. Different rock formations have different properties. When a landslide occurs, there will be some relative sliding between the different rock formations. We can distinguish the boundaries of different rock formations and the sliding zone from the data curves. The areas near 9 m and 57 m represent the boundary between earth-yellow clay with gravel and purplish-red gravel with clay on the tensile side (rear edge) of the anti-slide pile and compression side (front edge) of the anti-slide pile, respectively. Because the boundary between the loaded section and embedded section of the anti-slide pile is close to the boundary between strong and weak rock formations, the range of 16–18 m and 50–51 m is the optical data from the sliding zone to the boundary between strong and weak rock formations. They have stage differences, with the two regions fluctuating within their respective strain ranges. Because the pile plane size is much smaller than the pile length and the sensing fibers have several corners at the bottom of the pile, the strain data have abrupt changes at the bottom of the pile, as seen in the middle of the strain curves. Moreover, under the shear action of the sliding body, the strain value at the junction of the anti-slide pile and the sliding zone is in an abnormal state, which is reflected in the shadow strips in Figure 5, the strain value of this part is obviously different from that of other parts.

Thus, according to field records and data collected for sensing fibers, the corresponding tension side, pile bottom, and compression side of the anti-slide pile and the sliding zone can be identified in the four curve graphs of sensing fiber strain. Subsequent stress analysis can be aided by clearly identifying the corresponding positions of sensing fibers on the anti-slide pile.

**Figure 6:**
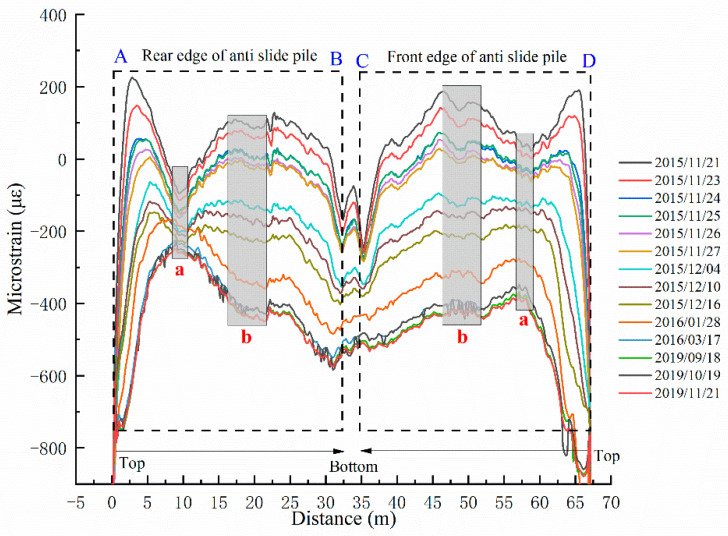
Temperature curves of MS-T1 for different periods. (In this figure, a and b represent the same meanings as in Figure 5).

It is worth noting that the temperature information described in the paper refers to the strain changes of the sensing fibers affected only by temperature, which can help eliminate the influence of temperature changes in the strain data. As Figure 6 illustrates, it is the temperature changes of the anti-slide pile in all periods. The temperature curves are symmetrical from about 0 m to 66 m, and the two symmetrical regions fluctuate in the same range. On the whole, the temperature of the anti-slide pile bottom is lower than that of the anti-slide pile body. Based on the curves and the actual situation, the temperature variations from 2015 to 2019 can be divided into three stages. In the initial stage, the temperature differences between the anti-pile body and bottom are distinct. In the middle stage, the temperature decreases as a whole, and the anti-pile bottom’s temperature curves become gentle. In the later stage, the anti-pile body and bottom data form a relatively stable curve, indicating that the temperature has stabilized. The data anomalies at the junction of the anti-slide pile and the sliding zone can be revealed by observing the temperature data curves. The abnormal temperature around the sliding zone indicates that the temperature at the junction of the anti-slide pile and the sliding zone will also be affected when the anti-slide pile is subjected to a large shear force.

The sensing fibers data monitored by BOTDA technology can observe the strain and temperature changes of different parts of the anti-slide pile. The strain information and temperature information can be used to locate any part of the anti-slide pile along the fibers, judging the force condition and safety of the anti-slide pile. The internal force characteristics and bending moment distribution of anti-slide piles can be analyzed using strain and temperature data in combination with other methods. Studying the anti-sliding effect of the anti-slide pile under the long-term action of landslides can help to understand the mechanism of action of anti-slide piles and the development trend of landslides.

#### 2.3.2. Data Comparison of Sensor Fiber and Steel Rebar Meters

**Figure 7:**
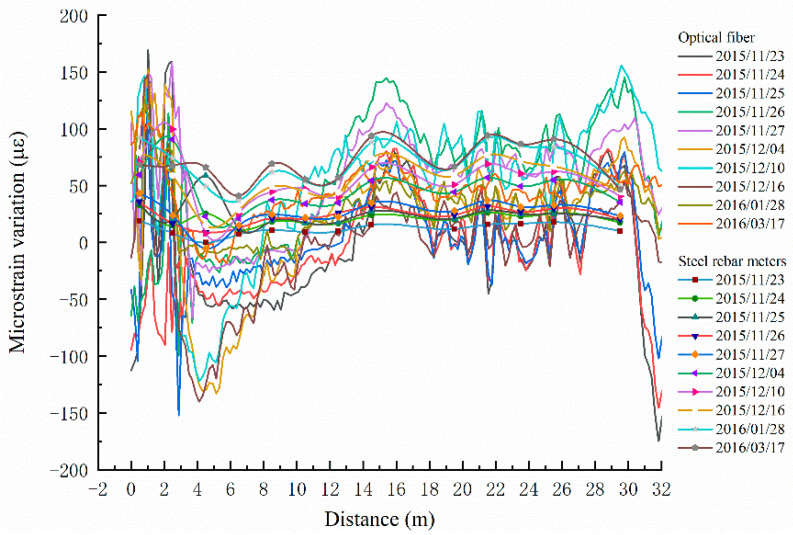
Comparison diagram of MS2 optical fiber and steel rebar meters.

Data from MS2 sensing fiber and steel rebar meters laid on the same reinforcement rebar were also analyzed. Taking the MS2 sensing optical fiber data on 21 November 2015 as the reference value, the differences between the 10 groups of data collected from 23 November 2015 to 17 March 2016 and the reference value are calculated. The strain variations caused by external factors are only considered. The strain variations caused by the anti-slide pile temperature do not need to be considered in internal force analysis. In view of this situation, each group of data needs to eliminate the influence of the temperature changes of the anti-slide pile described in Section 2.3.1. Thus, the strain changes described hereafter are caused simply by the anti-slide pile being pushed by the landslide. The steel rebar meter data use the same processing method. As Figure 7 shows, they are the strain curves obtained by sensing optical fiber and steel rebar meters, respectively. Among them, those with marks are the data curves monitored by steel rebar meters, while the others are the data curves of optical fiber.

The data show stress concentration at the bottom of the anti-slide pile, for the sensing fibers had several corners near the bottom. This phenomenon corresponds to the “stress bulges” near the 30 m depth in strain curves of the sensing fiber shown in Figure 7. What’s more, there are “bulges” around the depth of 15–16 m. Taken together with the actual situation of anti-slide pile embedding, this reveals that the depth of 15–16 m exactly corresponds to the overlap of sliding zones #1 and #2, shown in Figure 3. In other positions—that is, at depths of 17–31.5 m—the strain variations of the sensing fiber frequently fluctuate between −50 με and 150 με, which is to say that the strain data of the sensing fiber can accurately describe the position of the sliding zone and can also reflect the actual force of the anti-slide pile at any depth. In the strain change graph of steel rebar meters, the depth of the maximum strain change values does not absolutely correspond to that in the sensing fiber graph, owing to the lack of 15 m, 19 m, and 29 m depth data.

Nevertheless, the data of the sensing fiber and the steel rebar meter can reflect the force condition of the anti-slide pile. The steel rebar meter is a point-type data acquisition instrument, affecting its microstrain recognition ability. We collected the data from each steel rebar meter, but the variation of data from steel rebar meter to steel rebar meter was not available. If the steel rebar meter was damaged in some locations, the data losses would have a greater impact on the ability to identify microstrains, and the data will not reflect the true force situation. The data of any point on the sensing optical fiber can be obtained due to the dense sampling interval. On the strain change graphs, the data of the sensing fiber show more frequent volatility, whereas the data of the steel rebar meters show a smooth feature. This is because the sampling interval of the steel rebar meters is much larger than that of the sensing optical fiber. The fitting accuracy of the data between the sampling points of the steel rebar meters is much lower than that of the sensing optical fiber with dense sampling intervals.

In summary, the use of DOFS-BOTDA is the equivalent of placing hundreds or even thousands of monitoring points in the anti-slide pile. The number of measuring points in the anti-slide pile by steel rebar meters is limited, so monitoring data of steel rebar meters cannot adequately reflect the situation.

## 3. Analysis of Internal Force Characteristics of Anti-Slide Pile

### 3.1. Calculation Model of Anti-Slide Pile Internal Force

Because DOFS-BOTDA technology has the advantages of full distribution and high precision and does not interfere with temperature and strain measurement, the application of this technology to anti-slide pile deformation monitoring is highly significant. In this paper, the force condition of the anti-slide pile is analyzed and the bending moment distribution along the depth of the anti-slide pile is calculated and compared with the design value. The deformation characteristics of the anti-slide pile are assessed by evaluating whether the anti-slide pile works under normal stress conditions—a finding that is of practical significance to the study of anti-slide piles and landslides.

**Figure 8:**
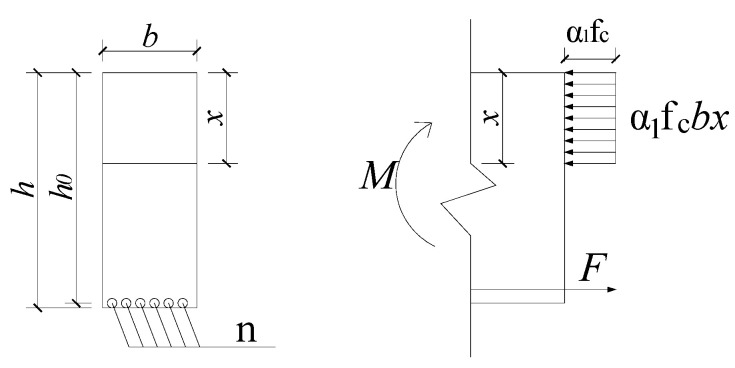
Schematic diagram of single reinforced rectangular normal section bending member model.

After obtaining the axial strain data of the anti-slide pile at different depths and times using the sensing optical fibers, mechanical equations are established according to the relationship between the strain and the force of longitudinal reinforcement in the anti-slide pile. The internal force of the anti-slide pile is also analyzed quantitatively. In this paper, a rectangular section bending member model is adopted, as shown in Figure 8, to calculate the internal forces of anti-slide piles. Based on previous research results, relevant calculations are carried out after the following assumptions for the model:

① The model satisfies the basic assumptions for calculating the bearing capacity of the normal section.
② The influence of the anti-slide pile self-weight stress is negligible.
③ Stress on the reinforcement bars on the compression side is ignored.
④ The stages of stress development of reinforced concrete are ignored.

The pure strain values of the bending member are obtained by subtracting the values of the temperature fibers from the values collected by the strain fibers. The stress σ at each point on the tensile side depth of the anti-slide pile can be calculated using the pure strain value ε and the elastic modulus E of the sensing fibers:

(12) $$\varepsilon =\varepsilon_{strain}-\varphi \varepsilon_{tem}$$

(13) σ=E∗ε

According to the theory of reinforced concrete structure and the principle of force balance, it can be seen that

(14) $$F=\sigma A_{s}n$$

Combining Equations (13) and (14) produces

(15) $$F=E\varepsilon A_{s}n$$

and

(16) $$F=\alpha_{1}f_{c}bx$$

According to the torque balance condition,

(17) $$M=F\left(h_{0}-x\right)$$

Substituting the F in Equation (15) and the x obtained from Equation (16) into Equation (17) produces

(18) $$M=E\varepsilon A_{s}n\left(h-\frac{E\varepsilon A_{s}n}{\alpha_{1}f_{c}b}\right)$$

where φ is the correction coefficient of strain, F the total tension of tensile reinforcement, $\alpha_{1}$ the equivalent rectangular stress coefficient of the concrete compression zone, $f_{c}$ the axial compressive strength of concrete, b the section width, x the height of the compression zone of the equivalent rectangular stress force, $h_{0}$ the effective height of the section, and M the bending moment of the bending member. The data described in the foregoing formulas have distributed characteristics because of the distributed nature of DOFS-BOTDA. Thus, the bending moment distribution at any point on the tensile side depth of the anti-slide pile can be obtained.

### 3.2. Internal Force Calculation of Anti-Slide Pile Based on DOFS

#### 3.2.1. Bending Moment Distribution

**Figure 9:**
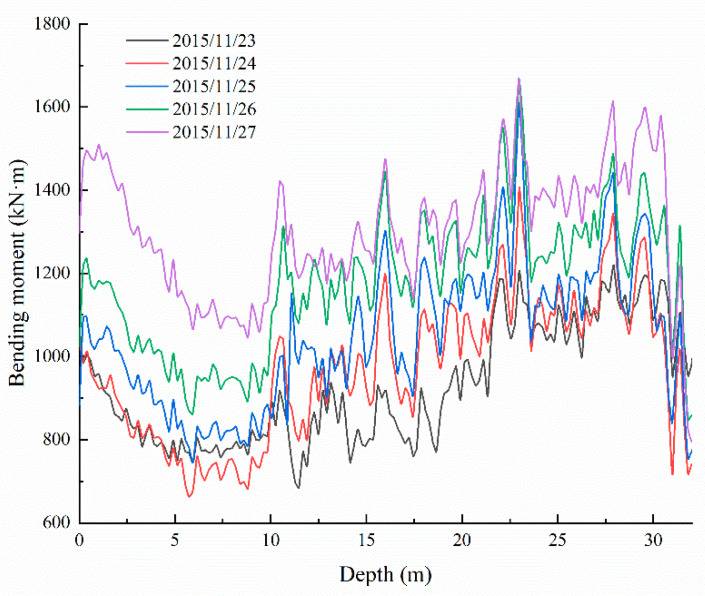
Early-stage graph of bending moment development of the anti-slide pile.

**Figure 10:**
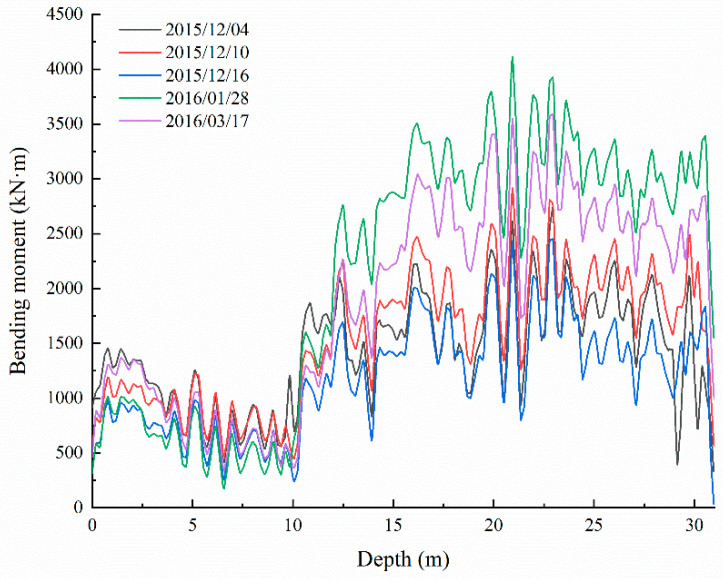
Second stage graph of bending moment development of the anti-slide pile.

**Figure 11:**
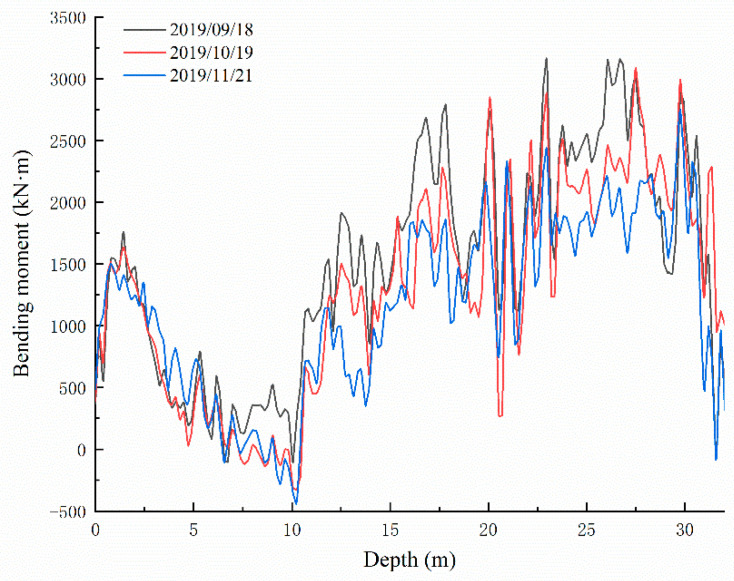
Third stage graph of bending moment development of the anti-slide pile.

Based on the preceding analysis, the bending moment distribution of the anti-slide pile in different periods can be calculated using the strain data obtained from the actual monitoring of the Sanzhong landslide, as shown in Figure 9, Figure 10 and Figure 11. The bending moments of the anti-slide pile can be divided into three stages, from 2015 to 2019. In the first stage, after the anti-slide pile casting is completed, the bending moment distribution along the depth is relatively gentle, during which only a large bending moment occurs at the lower part of the pile, as shown in Figure 9. At this stage, the maximum bending moment value does not exceed 1700 kN·m. According to the trend analysis, the bending moment value will continue to increase, indicating that the anti-slide pile has begun to play a role in the early stage of construction.

In the second stage, shown in Figure 10, the anti-slide pile continues to function. There is an obvious curve uplift in the figure. The maximum bending moment generated in this stage did not exceed 4200 kN·m, significantly more than in the first stage. At this stage, the maximum bending moment is located at the cross-section 20–24 m from the pile top. According to inspection records at the site, no new cracks, collapses, or deformations had been seen, indicating that the anti-slide pile had a strongly beneficial effect around 2016.

In 2019, data collection was carried out of sensing fibers in what can be classified as the third stage, as shown in Figure 11. During this stage, the bending moments distributed along the tensile side of the anti-slide pile still bulged. On the whole, the bending moment values in this stage were smaller than in the second stage but larger than in the first stage. The maximum bending moment along the pile body did not exceed 3100 kN·m, and the maximum values were also located at the cross-section 20–24 m from the pile top.

The foregoing three stages are trend analyses of the bending moment development of the anti-slide pile. All the monitoring time here is not the beginning or end time of each stage but included in the process of each stage. In other words, 14 monitoring periods between 2015 and 2019 identified these three stages but not the boundaries of the three stages.

#### 3.2.2. Fitting of Bending Moment

**Figure 12:**
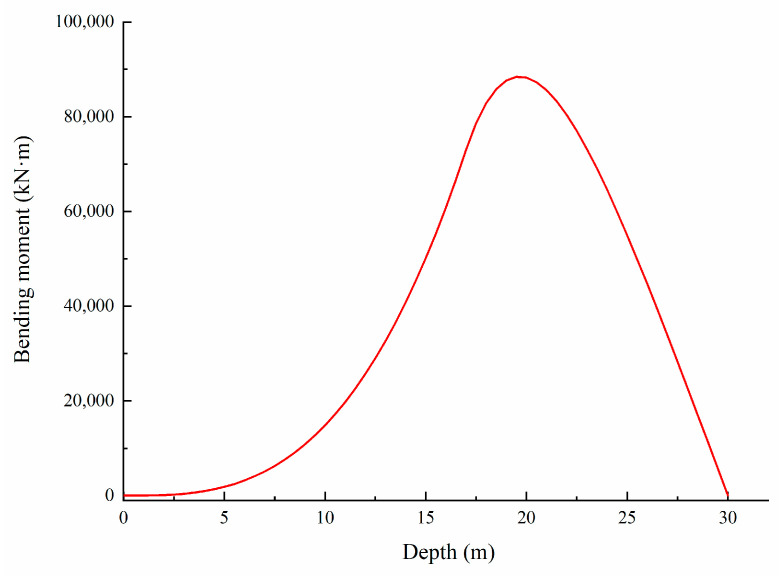
Designed bending moment distribution of the anti-slide pile.

**Figure 13:**
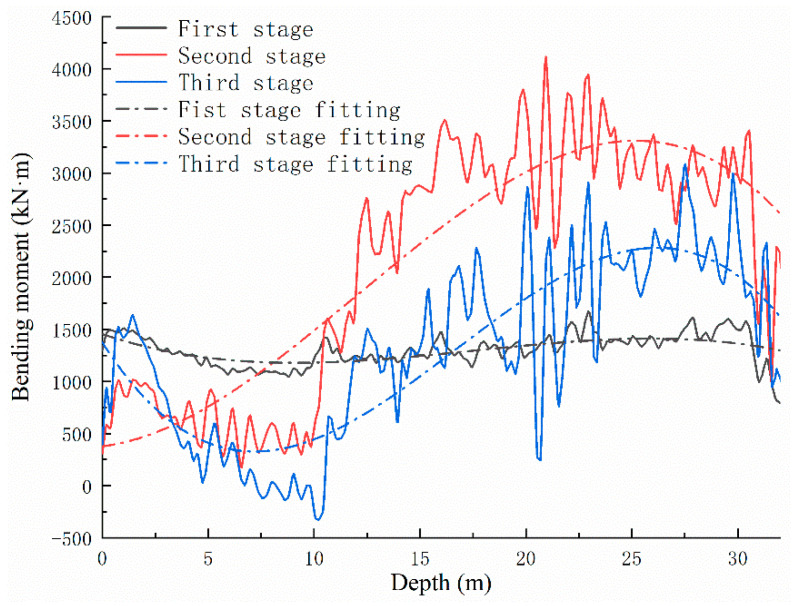
Graph of bending moment fitting of the anti-slide pile at different stages.

Reflecting relevant design requirements and data, the design bending moment distribution of the tensile side of the anti-slide pile is shown in Figure 12. The anti-slide pile force calculation adopts the K method, and the thrust distribution adopts the triangular model. It can be seen that the maximum bending moment value appears about 20 m from the top of the anti-slide pile in the designed bending moment distribution curve. The data sets with the larger bending moment distributed along the pile body in each stage were selected for curve fitting, as shown in Figure 13, based on analysis of the three stages of the anti-slide pile bending moment. It was found that the trend of fitting bending moment distribution curves was very close to that of the design bending moment distribution curve, with the calculated bending moment values far less than designed. In the three groups of bending moment fitting curve diagrams, the bending moment values near 0–6 m depth differ greatly from the designed ones. The bending moment fitting curves indicate that the positions from 31 m to 32 m belong to the bottom of the anti-slide pile. At the same time, it can be found that the maximum bending moments all appear in the vicinity of 22–23 m in the curves of bending moment fitting in the three stages.

Based on the actual situation, the possible reasons are as follows:

① Positions of 0–6 m are close to the top of the anti-slide pile, and all sensing fibers were pulled from this spot for monitoring. The closer they were to the anti-slide pile top, the more their data were affected by external factors. The calculated bending moment values were affected accordingly, producing incomplete and inaccurate fitting results.
② The lengths of the three rows of tensile reinforcement bars on the anti-slide pile differed, with tensile reinforcement bars spaced at 20 cm. When optical fibers crossed the two adjacent rows of reinforcement bars, they were usually wired diagonally downward, complicating the correspondence between the monitoring data and the depth of the anti-slide pile.
③ In the construction process, it was found that the actual sliding zone depth was 0.5–1 m deeper than the design. Thus, the actual 31.5 m length of the anti-slide pile was changed from 30 m in the original design, which impacted the actual bending moment distribution of the anti-slide pile.

In short, the bending moments of the anti-slide pile were still within the acceptable design range. This also shows that the landslide after control has been relatively stable. The strain and temperature data obtained by the DOFS-BOTDA system can accurately reflect the action process of the landslide thrust against the anti-slide pile within a certain range after processing and related mechanical calculations. It is essential to accurately determine the internal force distribution of the anti-slide pile and to judge its working state, which plays an important role in safety monitoring and early warning after landslide treatment.

### 3.3. Internal Force Training Model of the Anti-Slide Pile Based on Machine Learning

**Figure 14:**
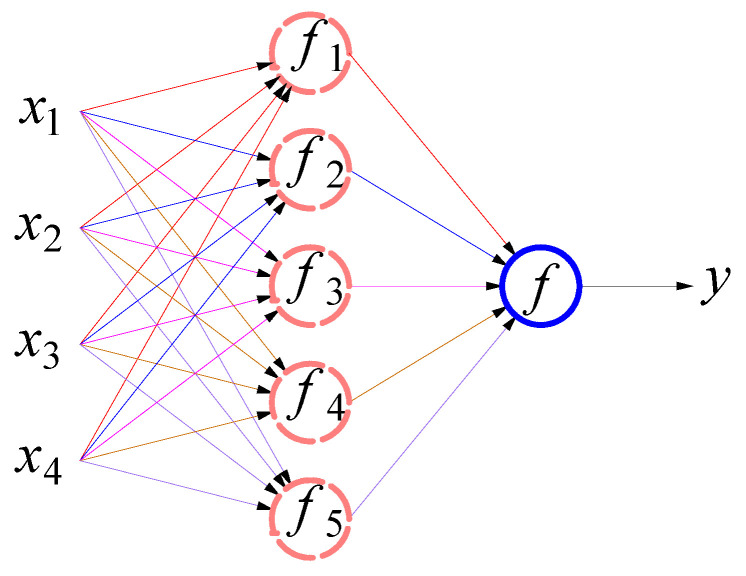
Process diagram of BP neural network.

**Figure 15:**
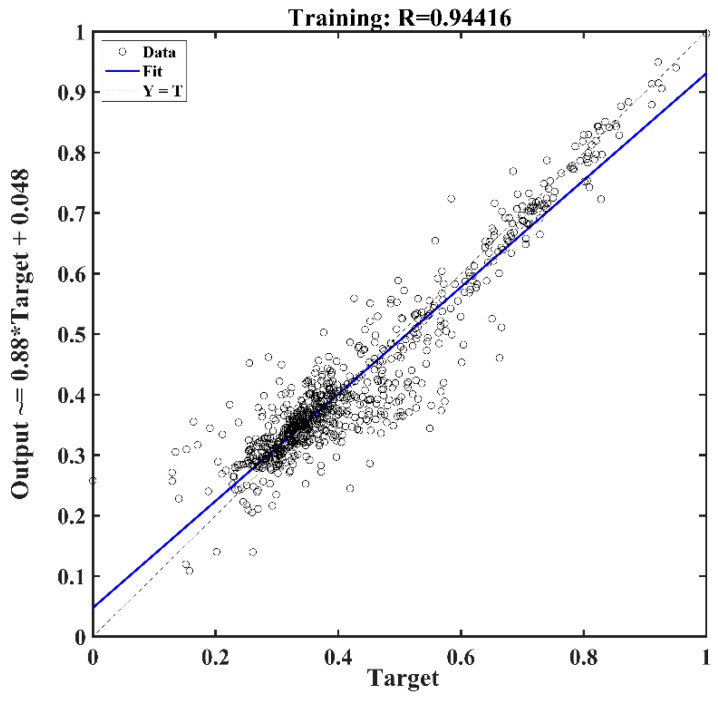
Correlation analysis diagram of training set model.

As Figure 14 shows, the BP neural network used in machine learning consists of input layers, hidden layers, and output layers, which enable the trained model to obtain the output values closest to the target values when given the input values. Based on the calculation process of the bending moment, depths of 10–33 m and the corresponding pure strain variations of the MS2, MS3, and MS5 sensing fibers are selected as input values, with the bending moment values used as the target values for data training. The pure strain values on 21 November 2015, are taken as initial values, with 13 sets of corresponding strain changes through 21 November 2019. This paper selects the first 80% of 13 data sets as the training set and the remaining 20% as the test set. The corresponding parameters of the neural network algorithm are set as follows: the maximum number of iterations 1000, target accuracy 0.001, learning rate 0.01, and 9 hidden layer nodes. After several pieces of training, the correlation coefficient graph of the training set in Figure 15 is shown. The correlation coefficient calculated from the training set’s output and target values is 0.9442. Most of the data in the training set are distributed along a 45° diagonal, indicating that the BP neural network model used for training had a good effect.

**Figure 16:**
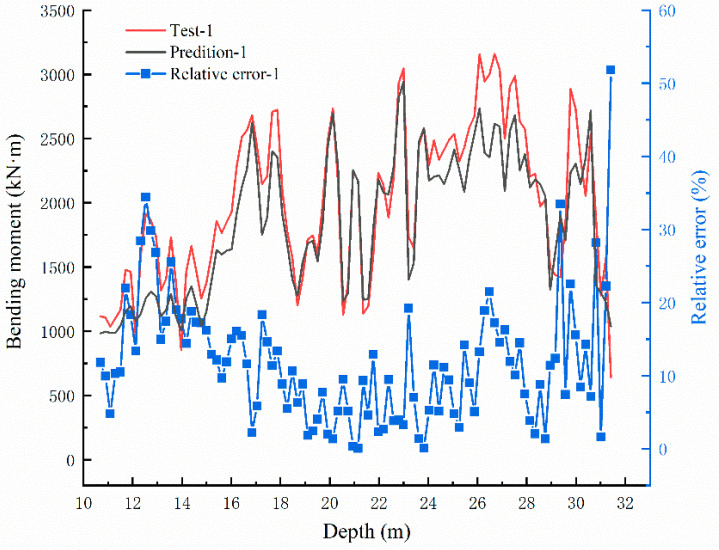
Bending moment comparison graph between Test-1 and prediction value and the error distribution curve.

**Figure 17:**
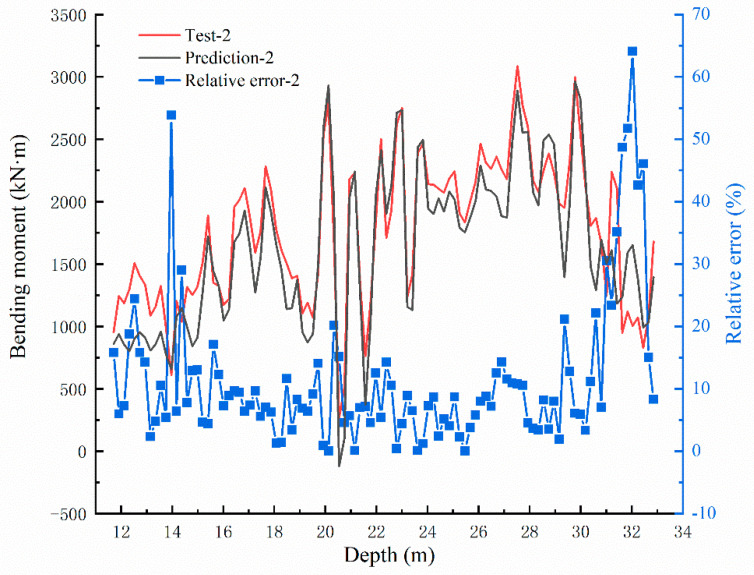
Bending moment comparison graph between Test-2 and prediction value and the error distribution curve.

**Figure 18:**
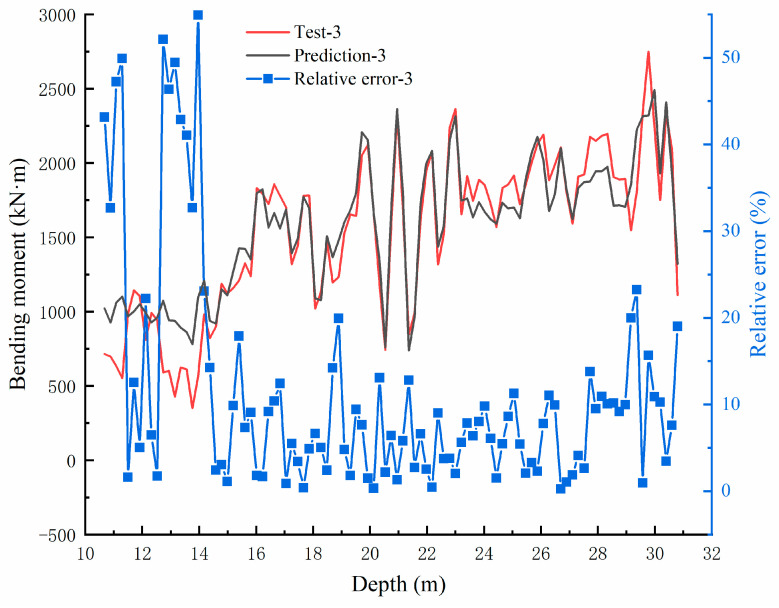
Bending moment comparison graph between Test-3 and prediction value and the error distribution curve.

The test results of bending moment values are compared with the original data, as shown in Figure 16, Figure 17 and Figure 18. In the three groups of comparison charts, the test data and prediction data maintain the same trend and are highly consistent. Using the determination coefficient formula,

(19) $$R^{2}=\frac{{\left(n\sum_{i=1}^{n}{\hat{y}}_{i}y_{i}-\sum_{i=1}^{n}{\hat{y}}_{i}\sum_{i=1}^{n}y_{i}\right)}^{2}}{\left(n\sum_{i=1}^{n}{\hat{y}}_{i}^{2}-{\left(\sum_{i=1}^{n}{\hat{y}}_{i}\right)}^{2}\right)\left(n\sum_{i=1}^{n}y_{i}^{2}-{\left(\sum_{i=1}^{n}y_{i}\right)}^{2}\right)}$$

where n is the number of test sets; ${\hat{y}}_{i}$ the output value; $y_{i}$ the target value; and i=1,2,3,……,n.

The determination coefficients of the three groups of predicted data can be calculated as 0.8506, 0.8734, and 0.8449, respectively, reflecting the strong correlation between the predicted data and the test data. The relative errors between the predicted and test values are calculated, and the error distribution curves are shown in Figure 16, Figure 17 and Figure 18. The minimum error fields in the relative error curves of each group are distributed in the range of 15–27 m, and the relative errors fluctuate within 10%. This range happens to be the relatively large range of bending moment values, showing that the BP neural network model designed in this paper can predict the bending moment of the anti-slide pile with good effect.

## 4. Results and Discussions

The steel rebar meter is a point-type data acquisition instrument, and its data reflect the strain and temperature information of the position for the steel rebar meter. The variations of data between each steel rebar meter can only be obtained by fitting. Its data graph is noticeably smooth, reflecting data distortion. It has the disadvantage of missing data for some important positions at this paper’s 2 m sampling interval, and the data curve is incomplete. It cannot reflect the actual strain and temperature information if the steel rebar meter is damaged at some locations.

The DOFS-BOTDA monitoring data can reflect the microstrain and temperature changes along the sensing fibers for the 0.2 m sampling interval in this paper. The data curves of sensing fibers have certain fluctuations, which reflect the actual strain and temperature information for any depth of the anti-slide pile. Due to the distributed characteristics, there is no risk of failing detection in the sensing fiber data. Compared with the data from the steel rebar meters, the sensing fibers can better reflect the force condition of the anti-slide pile while locating the sliding zone accurately. Thus, analysis of internal force characteristics of the anti-slide pile is more in line with the actual situation when using the sensing fiber data, which has more practical engineering significance for judging the stability of the anti-slide pile under the long-term effect of the landslide thrust.

During the three stages in which the anti-slide pile plays its role, the external force acting on the anti-slide pile gives rise to two processes, growth period and relative stability period, that are influenced by the soil behind the tension side of the anti-slide pile, among other factors. In view of the trend of the three stages of the anti-slide pile bending moment, it can be conjectured that the growth period is a change period of landslide force from a small level to a large level, which includes the first two stages of the bending moment development of the anti-slide pile described herein. The external force at the relative stability period is obviously less than that at the growth period. However, some other forces, such as the self-weight stress of soil, are inevitable, so the anti-slide pile is also subject to certain external forces during the relative stability period. The anti-slide piles can play a long-term anti-sliding role in landslides and can effectively control landslides to a great extent. However, it should be noted that if the strain that was in the relative stability period is found to have changed greatly in the later monitoring, corresponding investigations should be immediately carried out to determine whether the landslide enters a new growth period.

The unstable data segments are abandoned, and the data sets at 10–33 m, which are more in line with the actual situation, are selected for the BP neural network model training. The correlation coefficient, determination coefficient, and relative error between the values predicted by the training model and the actual values are calculated. According to the calculation results, the model has a good effect and is relatively stable. To some extent, it can predict the bending moment at any point of the anti-slide pile.

## 5. Conclusions

From the foregoing studies, we can draw the following conclusions:

(1) The effect of the anti-slide pile in landslide control has been verified. It can exert a long-term anti-sliding role by analyzing monitoring data, as seen in recent years. If other measures are combined in engineering practice, landslide control may reach its apex.
(2) DOFS technology based on BOTDA gives full rein to advantages of full distribution, high precision, and long distance in monitoring landslide anti-slide piles. This technology can collect strain and temperature data at any point along the optical fiber in a structure at a given time. The collected data can be processed twice to extend it in the needed direction.
(3) The bending moment calculation model of the anti-slide pile is established by the monitoring data from sensing fibers in the anti-slide pile. Using this model, the bending moment distribution along the anti-slide pile and its distribution form can be obtained. The internal force state of the anti-slide pile is analyzed qualitatively and evaluated quantitatively based on monitoring data obtained from the sensing fibers. The design values and calculation values of the internal force of the anti-slide pile are compared and analyzed, and the working state of the anti-slide pile is evaluated. At present, the landslide is in a stable state, and the anti-slide pile still plays a good effect in the anti-sliding function.
(4) The BP neural network used in machine learning can be used to predict the bending moments of anti-slide piles. After several training iterations, a neural network model that reflects the mapping relationship between input sets and output sets can be obtained by selecting appropriate input sets and their corresponding output sets. The BP neural network model can provide a novel data analysis and prediction method for future engineering monitoring, combining engineering building and monitoring methods.

This study does have certain limitations, however. Due to the different lengths of the three rows of tensile reinforcement bars, sensor fibers were not deployed entirely according to the theoretical design, leading to certain data processing and analysis errors. To prevent overfitting, 10 training sets and 3 test sets were used to train the BP neural network model. Still, data underfitting may occur in BP neural network training, as reflected in comparative analysis between predicted data values and the actual data values in this paper.
